# Effects of Preoperative Abnormality of Posterior Tibial Tendon on the Surgical Outcomes of Medial Osteochondral Lesion of the Talus

**DOI:** 10.7759/cureus.62046

**Published:** 2024-06-10

**Authors:** Shingo Kawabata, Tomoyuki Nakasa, Yasunari Ikuta, Satoru Sakurai, Dan Moriwaki, Saori Ishibashi, Nobuo Adachi

**Affiliations:** 1 Department of Orthopedic Surgery, Graduate School of Biomedical and Health Sciences, Hiroshima University, Hiroshima, JPN; 2 Department of Artificial Joints and Biomaterials, Graduate School of Biomedical and Health Sciences, Hiroshima University, Hiroshima, JPN

**Keywords:** flatfoot, mri, posterior tibial tendon, talus, osteochondral lesion

## Abstract

Background: Although surgical treatment for osteochondral lesion of the talus (OLT) can obtain good clinical outcomes, the rate of return to sports is variable. It is reported that medial OLT unrelated to trauma has abnormal structures in the medial aspect, which may induce the medial OLT due to the medial instability. The posterior tibial tendon (PTT) plays an important role in the stabilization of the foot, and high mechanical stress may be added to the PTT to compensate for medial instability in medial OLT. We investigated whether abnormal PTT findings on preoperative magnetic resonance imaging (MRI) in patients with OLT affect clinical outcomes after surgery.

Methods: Eighty-one ankles in 74 patients who were treated surgically for OLT were included in this study (41 men and 33 women; mean age, 26.0 years). Abnormalities of the PTT were evaluated using preoperative MRI. The Japanese Society for Surgery of the Foot (JSSF) scale, arch height, and ankle activity score (AAS) on standing plain radiogram were compared between patients with and those without preoperative PTT abnormalities.

Results: Twenty-five ankles (30.9%) had PTT abnormalities on preoperative MRI. All patients with preoperative PTT abnormalities were medial OLT. There were no significant differences in the preoperative JSSF scale in the procedures for OLT. The postoperative JSSF scale and arch height were significantly lower in patients with preoperative PTT abnormalities than those without them. AAS in patients with preoperative abnormalities significantly decreased at the final follow-up.

Conclusion: PTT abnormalities on preoperative MRI may affect clinical outcomes even in preoperative asymptomatic patients in the medial OLT unrelated to trauma.

## Introduction

An osteochondral lesion of the talus (OLT) is an intra-articular lesion that affects the articular cartilage and subchondral bone. Because of the low success rate of conservative treatment in our population in the risk of osteoarthritis, OLT often requires surgical treatment [1]. Although trauma is recognized as the main cause of OLT, several factors unrelated to trauma may also result in OLT [2,3]. The majority of the OLTs are located in the posteromedial and centromedial zone, and 70% of medial OLTs and 98% of lateral OLTs are associated with trauma, which suggests that several medial OLT cases are unrelated to trauma [4]. Notably, abnormal morphologies such as bone and ligament at the medial region of the ankle and medial instability in medial OLT have been reported [5,6]. These abnormal medial structures of the ankle and foot may affect the pathogenesis of the medial OLT. Patients with OLT complain of non-specific chronic ankle pain, without tenderness or pain that corresponds to the location of the OLT despite the existence of a focal lesion, and these nonspecific pains may be due to medial abnormal structures [1]. Previous studies have demonstrated abnormalities of the posterior tibial tendon (PTT), spring ligaments, and plantar fascia which are structures for foot stabilization on magnetic resonance imaging (MRI) [7]. Instability or laxity of these structures may cause medial pain due to damage to the longitudinal arch. Such abnormalities in the medial structure of the medial OLT may affect the clinical outcomes, even if the OLT has been completely healed. We hypothesized that PTT and spring ligament abnormalities possibly exist preoperatively in patients with medial OLT. These abnormalities may affect the postoperative clinical outcomes, especially in patients with medial OLT. This study aimed to assess whether the abnormal findings of PTT and spring ligament on preoperative MRI in patients with medial OLT might affect clinical outcomes after surgery.

## Materials and methods

Participants

Eighty-one ankles of 74 patients who were treated surgically for OLT between January 2006 and October 2020 were retrospectively reviewed. Patients with OLT who had preoperative MRI and had been followed up for at least one year after surgery were included in this study. Patients with chronic lateral ankle instability, and systemic diseases, such as rheumatoid arthritis, were excluded. Seven patients had bilateral ankles OLT. This study was approved by the local ethical committee of Hiroshima University (E-879), and informed consent was obtained from all individual participants included in this study.

MRI evaluation

Preoperative MRI scans were performed using a Signa 1.5-T device or a Signa HDxT 3.0-T device (GE Yokogawa Medical Systems Ltd., Tokyo, Japan) with a wraparound surface coil designed for the ankle joint. The ankles were fixed at a slightly plantarflexed position to minimize the magic angle artifact. Proton density spin-echo (SE) and T2-weighted SE images were acquired. The conditions for the T2-weighed images were as follows: repetition time, 2600 ms; echo time, 98 ms; and section thickness, 3.0 mm. The conditions for proton-weighted images were the following: repetition time, 2000 ms; echo time, 20 ms; and section thickness, 3.0 mm. The posterior tibial tendon and spring ligament were assessed on sagittal and axial MRI. OLTs were classified according to Anderson’s classification [8].

**Figure 1:**
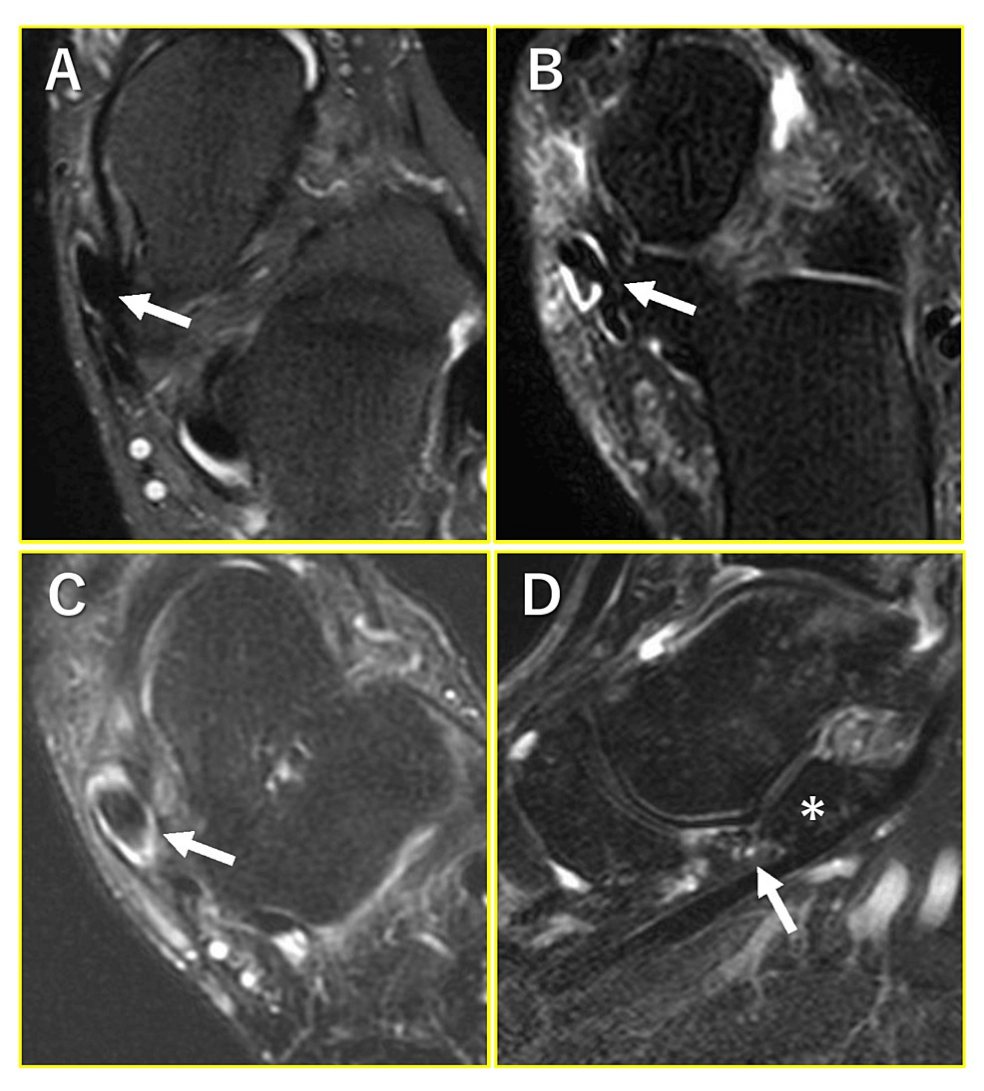
Representative MRI images of the posterior tibial tendon (PTT) and spring ligament. (A) Normal PTT. (B) Paratendonitis of PTT. (C) Tendonitis of PTT. The the arrows indicate PTT. (D) Abnormal finding of the spring ligament. The arrow indicates the spring ligament and the asterisk (*) indicates sustentaculum tali.

A normal PTT was described as black without any internal signal intensity, with a smooth curve around the medial malleolus limiting focal compression and impingement, and with a size that is roughly twice that of the flexor digitorum longus and flexor hallucis longus (Figure 1A) [9,10]. Abnormal findings of the PTT were classified as paratendonitis, tendonitis, and accessory navicular. Paratendonitis was defined as partially circumferential high signal intensity located distally around the PTT, although its signal intensity is slightly hypointense compared to that of fluids (Figure 1B) [10]. Tendonitis was defined as a subtle focal high signal intensity in the tendon, tendon thickening due to scaring by partial tears, and tendon thinning due to retraction by partial tears (Figure 1C) [10]. Abnormalities of the spring ligament were assessed on the sagittal images. Spring ligament injury was defined as thickened, disrupted, heterogeneous, or abnormal contour with attenuated signal intensity, according to a previous report (Figure 1D) [11]. Abnormalities of the PTT and spring ligament on MRI were evaluated by an experienced orthopedic surgeon (T.N.) with 20 years of relevant experience.

Radiographic evaluation

For the radiographic evaluation of the medial arch, lateral talo-first metatarsal angle (T1stMTA), calcaneal pitch angle (CPA), and medial cuneiform-fifth metatarsal height (MC5thMH) were measured on lateral weight-bearing images. T1stMTA was defined as the angle between the talar midline axis and a line that bisects the first metatarsal. A T1stMTA of more than 4° was defined as flatfoot [12]. The CPA was defined as the angle between the lines drawn along the inferior border of the calcaneus and the plantar aspect of the soft tissue of the foot, and MC5MH was the distance of the perpendicular lines extending to the floor between the most inferior portion of the distal aspect of the medial cuneiform and the base of the fifth metatarsal.

Assessment of clinical outcomes

Clinical outcomes before surgery and at the final follow-up were evaluated by the Japanese Society for Surgery of the Foot (JSSF) ankle/hindfoot rating scale [13,14] and ankle activity score (AAS) which consisted of 53 sports, three working activities, and four general activities [15]. In addition, residual symptoms described in the medical records were investigated at the final follow-up.

Operative procedure

Standard anterolateral (AL) and anteromedial (AM) portals were made, and a 30-degree oblique arthroscope was introduced. Anterior talofibular ligament (ATFL) was observed arthroscopically, and OLT patients with intact ATFL were included in this series. Based on arthroscopic findings, osteochondral fragments with International Cartilage Repair Society (ICRS) grade 1 or 2 were considered to be preserved. Retrograde drilling was performed for stable lesions, and fixation of fragments was performed using absorbable pins for unstable lesions. For the fixation of the osteochondral fragment, poly-L-lactide pins with a diameter of 2 mm or 1.5 mm were inserted into the osteochondral fragment until a rigid and stable construct was obtained. Osteotomy of the medial malleolus was carried out to approach the medial lesions. A lesion size less than 150 mm2 and osteochondral fragment with ICRS grade 3 or 4 was determined to be excised. Nevertheless, even if the ICRS grade was 1 or 2, a small lesion of approximately 50 mm2 was also excised. Microfracture was performed after the excision of the osteochondral fragment. For subchondral cysts with good cartilage surface, bone grafting was performed. The cartilage fragment above the subchondral cyst was elevated, and the cyst was curetted. Subsequently, bone grafting from the distal tibial metaphysis was performed. Finally, the cartilage fragment was fixed with 5-0 nylon sutures to the surrounding cartilage. Autologous osteochondral grafts from the knee joint were applied for subchondral cysts with a severely damaged cartilage surface.

Statistical analysis

Statistical differences between the two groups were calculated using the paired t-test for the comparison pre- and postoperative JSSF scales, the Mann-Whitney U test for the comparison of the JSSF scales between those with and those without medial malleolar osteotomy at the final follow-up, and the JSSF scales and radiographic parameters between normal and abnormal group. The Kruskal-Wallis test was used for multiple comparisons of the JSSF scale between operative procedures. P value of <0.05 was considered statistically significant.

## Results

Clinical outcomes

Patients consisted of 41 males and 33 females with a mean age of 26.0 years (range, 11-70 years). Medial lesions were found in 66 ankles, lateral lesions in 13 ankles, and central lesions in two ankles. These lesions were diagnosed as stage 2 in two ankles, stage 2A in 15 ankles, stage 3 in 58 ankles, and stage 4 in six ankles on MRI, according to Anderson’s classification. Operative procedures were as follows: excision of the osteochondral fragment and microfracture in 20 ankles, retrograde drilling in 11 ankles, fixation of the osteochondral fragment in 32 ankles, bone grafting for stage 2A lesion in 12 ankles, and autologous osteochondral graft in six ankles. Medial malleolar osteotomy for posteromedial lesion was required in 33 ankles and ankle lateral ligament repair in four ankles was performed. The mean follow-up period was 22.3 months (range, 12-102 months). The JSSF scale significantly improved from 71.9 points (range, 47-87 points) preoperatively to 95.8 points (range, 67-100) at the final follow-up (P<0.01). At the final follow-up, 29 ankles had residual pain around the ankle joint. Details of the symptoms are shown in Table 1.

**Table 1:** Residual symptoms at the final follow-up. OLT: Osteochondral lesion of the talus; PTT: Posterior tibial tendon.

Symptoms	Number (location of OLT)
Occasional pain at medial aspect	7 (medial)
Occasional pain at PTT	4 (medial)
Occasional pain at PTT after sports activity	4 (medial)
Occasional pain at lateral aspect	2 (medial, lateral)
Occasional pain at center of the ankle during walking	2 (center)
Pain at medial aspect during sports activity	1 (medial)
Severe pain at PTT	1 (medial)
Starting pain at medial aspect when walking	1 (medial)
Pain at medial and lateral aspect on tiptoe	1 (medial)
Pain at medial aspect when crouching	1 (medial)
Pain at lateral aspect during dorsiflexion	1 (lateral)
Pain at sinus tarsi	1 (lateral)
Occasional pain at lateral aspect on the stairs	1 (lateral)
Occasional pain at lateral aspect after long-distance walking	1 (lateral)
Starting pain at lateral aspect when walking	1 (lateral)

**Figure 2:**
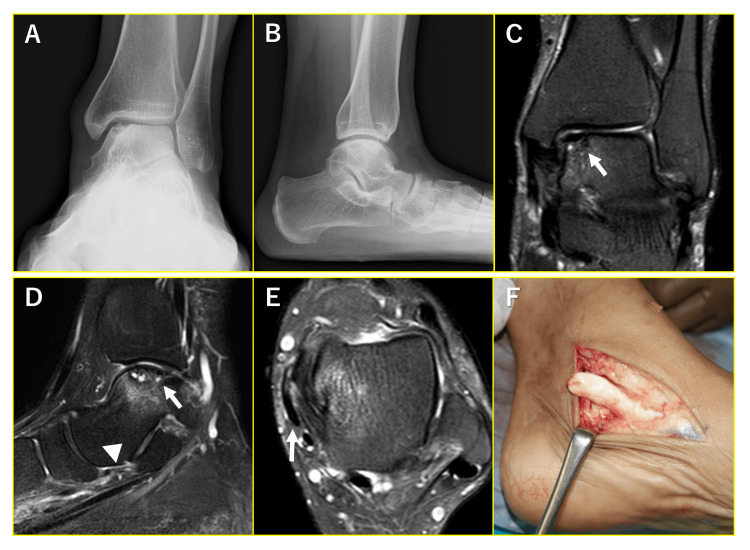
Posterior tibial tendon (PTT) rupture after the fixation of the osteochondral fragment. (A, B) Preoperative radiograms showing medial osteochondral lesion of the talus (OLT) and flatfoot deformity. (C, D) Preoperative MRI (coronal and sagittal images). Arrows indicate OLT and the arrow head indicates the spring ligament with thinning and alteration of signal intensity. (E) Preoperative MRI (axial image). The arrow indicates flattening of PTT. (F) Intraoperative findings of PTT rupture at seven months after OLT surgery.

Of the 29 ankles, 20 had operative treatment for medial OLT, of which two ankles exhibited severe pain at the PTT. One patient who underwent osteochondral fragment fixation with oblique medial malleolar osteotomy complained of severe pain which restricted the activities of daily living. Therefore, medial displacement calcaneal osteotomy and flexor digitorum longus tendon transfer were performed at seven months because of the PTT tear (Figure 2).

Other patients who underwent osteochondral fragment fixation without oblique medial malleolar osteotomy could not return to sports activity because of severe pain at the PTT. The JSSF scales at the final follow-up were not significantly different between the operative procedures (Table 2). In addition, there was no significant difference in the JSSF scale between those with and those without medial malleolar osteotomy.

**Table 2:** Follow-up period and JSSF scales in each operative procedure. JSSF: Japanese Society for Surgery of the Foot.

Procedure (number)	Follow-up period (range)	JSSF scale	
		Preoperative (range)	Final follow-up (range)
Fixation of osteochondral fragment (32)	19.3±9.0 months (12-48)	73.4±3.1 points (69-77)	97.2±6.8 points (67-100)
Excision with microfracture (20)	22.4±13.1 months (12-53)	72.0±4.5 points (66-87)	96.7±5.2 points (87-100)
Bone grafting (12)	29.7±22.6 months (12-93)	63.8±9.7 points (47-72)	90.6±6.1 points (77-100)
Autologous osteochondral graft (6)	28.2±13.7 months (14-49)	71.3±2.8 points (67-74)	94.0±5.7 points (87-100)
Retrograde drilling (11)	22.0±26.8 months (12-102)	74.6±5.2 points (69-83)	96.4±5.0 points (87-100)

MRI findings

Twenty-five ankles (30.9%) had abnormal findings of PTT on MRI: 12 ankles showed paratendonitis, 10 ankles showed tendonitis, and three ankles had accessory navicular. Moreover, 28 ankles (34.6%) had abnormal spring ligament findings: 13 ankles showed attenuated signal intensity, 13 ankles had abnormal contour, and two ankles showed thickened ligaments. All these MRI findings were observed in medial OLT.

Relationship between symptoms at the final follow-up and PTT abnormalities on preoperative MRI

**Figure 3:**
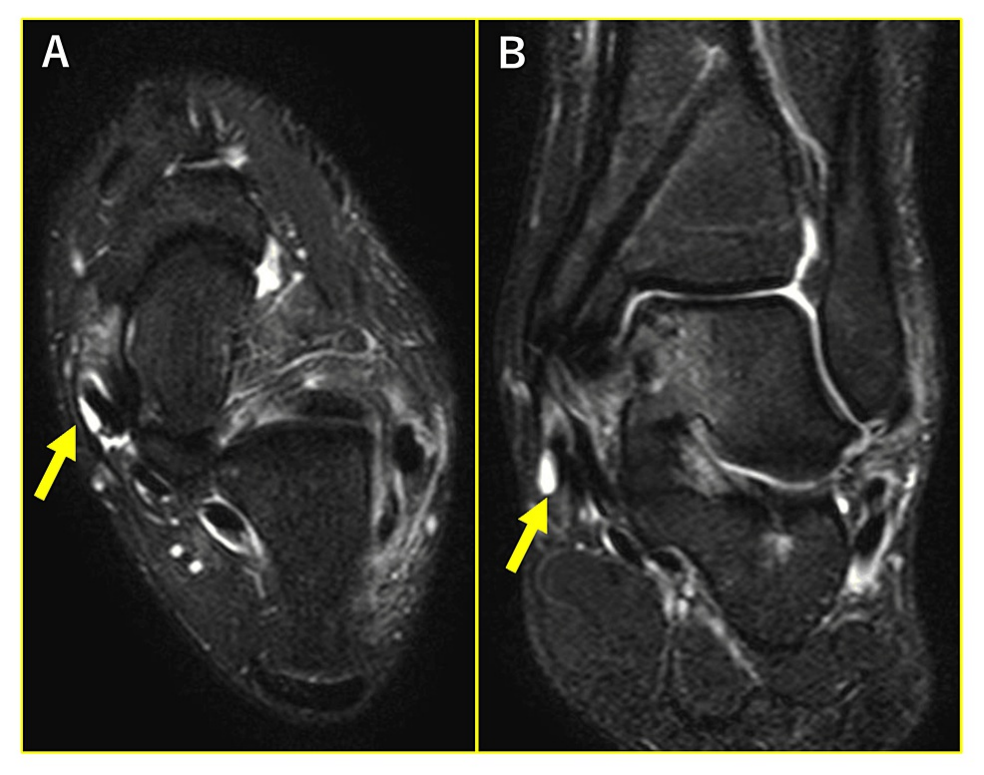
Representative images of posterior tibial tendon (PTT) abnormalities on preoperative and one-year postoperative MRI. (A) Preoperative MRI. (B) One-year postoperative MRI. Arrows indicate PTT abnormality.

In 20 ankles with medial pain, 13 had abnormalities of PTT (65%), two had accessory navicular (10%), and 13 had abnormalities of the spring ligament (65%) on preoperative MRI. Moreover, 11 ankles (55%) had both PTT and spring ligament abnormalities on MRI. Ankles with medial OLT (66 ankles) were divided into two groups according to the presence or absence of abnormalities of PTT on MRI: normal group and abnormal group. The normal group consisted of 24 ankles in 22 males and 17 ankles in 16 females (mean age, 24.1 years; range, 11-62 years). The abnormal group consisted of eight ankles in eight males and 17 ankles in 16 females (mean age, 27.4 years; range, 13-70 years) (Figure 3).

No significant differences in the follow-up period and preoperative JSSF scale were found, but the JSSF scale at the final follow-up in the abnormal group (93.3 points; range, 67-100 points) was significantly worse than that in the normal group (98.2 points; range, 87-100 points) (P<0.05). Regarding foot alignment, no significant differences in T1stMTA and MC5thMTH between the groups were observed. However, MC5thMTH in the abnormal group was significantly lower than that in the normal group (P<0.05). The results of the comparison between groups are shown in Table 3.

**Table 3:** Comparison between the normal and abnormal groups. JSSF: Japanese Society for Surgery of the Foot; T1stMTA: Talo-first metatarsal angle; CPA: Calcaneal pitch angle; MC5thMH: Medial cuneiform-fifth metatarsal height. * P<0.05.

Groups	Mean age (range)	Preoperative JSSF scale (range)	Postoperative JSSF scale (range)	T1stMTA	CPA	MC5thMTH
Normal group (n=41)	24.1 years (11-62)	72.4 years (47-87)	98.2 points (87-100)*	11.9° (-5.4-26.3°）	16.9° (8.2-21.6°）	6.5 mm (-2.1-13.7)*
Abnormal group (n=25)	27.4 years (13-70)	70.9 years (51-77)	93.3 points (67-100)	15.2° (-5.2-25.5°）	15.5° (3.4-26.9°）	4.5 mm (-4.7-13.5)

Relationship between preoperative abnormalities of PTT and sports activity

Fifty-three ankles in 50 patients had sports activity, and preoperative PTT abnormalities were found in 20 ankles. There were no significant differences in JSSF scales at final follow-up and AAS at preoperative and final follow-up between abnormal (n=20) and normal group (n=37). However, AAS at preoperative significantly decreased at the final follow-up. 

## Discussion

Our study revealed that abnormalities of PTT and spring ligament may exist in medial OLT, which could cause residual pain around the medial aspect of the ankle after surgery in medial OLT. Most of the symptoms observed at the final follow-up were minor. However, two patients had fairly severe pain, and one of them had a PTT rupture during the postoperative course. PTT and spring ligament abnormalities in the OLT should be taken into account in the OLT treatment to improve the clinical outcome.

Pain in OLT is considered to originate from the subchondral bone because OLT does not generally have much synovitis [16]. Increasing nerve endings in the subchondral bone are recognized as a factor that causes pain. In OLT, high-pressurized fluid flows into the subchondral bone through the fissure of the subchondral bone plate, which activates osteoclasts and macrophages. Activation of these cells may result in low pH, local bone demineralization, and H+-mediated stimulation of the primary afferent nociceptive nerve fibers in the subchondral bone [17]. In OLT, the area in the subchondral bone in which the joint fluid flows causes pain. However, in our study, the subchondral bone was unlikely to be the cause of pain at the final follow-up. No significant differences in clinical outcomes between operative procedures and between those with and those without medial malleolar osteotomy were found. Similarly, in a previous study, no significant difference in the clinical score at one year between patients with OLT with and without an osteotomy was found [18]. Thus, extra-articular medial structure, including PTT and spring ligament, could be the cause of pain after successful operative treatment of OLT.

The rate of return to sports activity after surgery and preoperative levels of sports are variable. A systemic review of autologous osteochondral transplantation for OLT showed that the mean rate of return to play was 86.3%, (range 50-95.2%) [19]. Previous studies demonstrated that in the bone marrow stimulation technique, the rate of return to any level of sports was from 77 to 88% and that of return to pre-injury level of sports was 79% [20,21]. Nevertheless, in a previous investigation, even if the return to sports was achieved, the activity level decreased at the long-term follow-up [22]. Although several reports have demonstrated the rate of return to sports among athletes with OLT, the factors that inhibit return to sports remain unclear. PTT and spring ligament abnormalities, which may exist preoperatively due to some predisposing factors such as medial and lateral instability or morphological abnormalities, may affect clinical outcomes, including return to sports activity. In this study, patients with preoperative PTT abnormalities decreased ankle activity scores at the final follow-up. Preoperative PTT abnormalities may be one of the predisposing factors to lower rates of return to sports and to the preinjury level of sports.

PTT dysfunction has a high incidence in the general population (5% to 15%), especially in women [23,24]. Nonetheless, subclinical tendinopathy of PTT is only identified by imaging or deformity without any clinical symptoms and can progress to a painful and debilitating condition, although some cases do not worsen to a clinically significant problem. In our series, 30.9% of patients with OLT had abnormal findings of PTT, and all of them had medial OLT. In addition, the MC5thMH was significantly lower in the patients with abnormal findings of PTT than in those with normal findings of PTT. Since our series had juvenile patients, the mean age was relatively younger for adult-acquired flatfoot deformity. Thus, a flatfoot with medial OLT in our series may be a flexible pediatric flatfoot. A previous report demonstrated that the depression of the foot arch was commonly identified in pediatric patients with medial OLT [3]. Symptomatic pediatric flatfeet may eventually progress to symptomatic adult flatfeet, which suggests that pediatric flatfoot potentially affects the PTT [25]. In our study, patients with medial OLT had more flatfeet, thus, the pathogenesis of medial OLT without trauma may be related to flatfeet. In patients with PTT dysfunction, plantarflexion and eversion of the hindfoot are increased [26]. Furthermore, a previous report has shown that the deep deltoid ligament at the talus is broader and located more proximally than the non-medial OLT, which may be affected by excessive stress to the posteromedial region of the talus [8]. The deep deltoid ligament is recognized as the primary restraint to lateral and posterior translation of the talus, valgus stress, internal rotation of the talus, and dorsiflexion of the ankle [27]. Therefore, flatfeet may provide excessive force to the deltoid ligament, which could in turn result in medial OLT. In addition to PTT, the spring ligament is an important component of the static restraints of the medial longitudinal arch of the foot. The superomedial ligament, which is composed of the spring ligament, connects to the medial malleolus as the tibiospring ligament [28]. A previous report has shown that the tibiospring ligament is under the most strain during eversion [25]. In flatfeet with increased eversion, there may be abnormalities in the spring ligament that are associated with the high strain of the tibiospring ligament. One of the challenges in the operative treatment of OLT is the absence of a postoperative rehabilitation protocol [7,29]. Treatment or postoperative rehabilitation of OLT must consider the aforementioned pathological conditions.

This study has several limitations. First, this study’s retrospective design may introduce certain bias, such as selection and recall bias, which could affect outcomes. Second, it is important to acknowledge the limitations associated with MRI accuracy. Previous studies have indicated that the diagnostic accuracy for detecting pathologies of the PTT is 96% [30]. This factor should be considered when interpreting the findings from our MRI assessments. Third, the number of lateral OLTs was smaller than that of medial OLTs. Although all patients with abnormal findings of PTT on MRI had medial OLT, there may be patients with PTT abnormalities among those with lateral OLT. In addition, whether pain was caused by the PTT in all patients was not confirmed, although some patients had obvious pain in PTT, some patients complained of medial side pain at the final follow-up. No difference in the clinical score between operative procedures, including medial malleolar osteotomy, was found, and no failure of the lesion based on the image findings was observed. Thus, the cause of pain in the medial aspect could be the PTT. Prospective studies using a large number of patients are needed to explore the effect of PTT on the clinical outcome of OLT treatment.

## Conclusions

This study demonstrated that posterior tibial tendon (PTT) abnormalities on preoperative MRI may affect the clinical outcome of the operative treatment of osteochondral lesions of the talus (OLT). Potential tendinopathy of PTT may be identified only by imaging or deformity without clinical symptoms and may progress to a painful and debilitating condition, although in some cases it may not worsen to a clinically significant problem. Postoperative rehabilitation must consider PTT abnormalities to improve the clinical outcome of OLT treatment.

## References

[1] Gianakos AL, Yasui Y, Hannon CP, Kennedy JG (2017). Current management of talar osteochondral lesions. World J Orthop.

[2] Berndt AL, Harty M (2004). Transchondral fractures (osteochondritis dissecans) of the talus. J Bone Joint Surg Am.

[3] Ikuta Y, Nakasa T, Sumii J, Nekomoto A, Adachi N (2023). Radiographic foot alignment and morphological features of deltoid ligament in pediatric patients with medial osteochondral lesions of the talus. J Pediatr Orthop B.

[4] van Diepen PR, Dahmen J, Altink JN, Stufkens SA, Kerkhoffs GM (2021). Location distribution of 2,087 osteochondral lesions of the talus. Cartilage.

[5] Nakasa T, Sawa M, Ikuta Y, Yoshikawa M, Tsuyuguchi Y, Adachi N (2018). Anatomic feature of deltoid ligament attachment in posteromedial osteochondral lesion of talar dome. J Orthop Sci.

[6] Teramoto A, Shoji H, Kura H, Sakakibara Y, Kamiya T, Watanabe K, Yamashita T (2018). Investigation of factors related to the occurrence of osteochondral lesions of the talus by 3D bone morphology of the ankle. Bone Joint J.

[7] Koç A, Karabiyik Ö (2018). MRI evaluation of ligaments and tendons of foot arch in talar dome osteochondral lesions. Acta Radiol.

[8] Anderson IF, Crichton KJ, Grattan-Smith T, Cooper RA, Brazier D (1989). Osteochondral fractures of the dome of the talus. J Bone Joint Surg Am.

[9] Khoury NJ, el-Khoury GY, Saltzman CL, Brandser EA (1996). MR imaging of posterior tibial tendon dysfunction. AJR Am J Roentgenol.

[10] Schweitzer ME, Karasick D (2000). MR imaging of disorders of the posterior tibialis tendon. AJR Am J Roentgenol.

[11] Perrich KD, Goodwin DW, Hecht PJ, Cheung Y (2009). Ankle ligaments on MRI: appearance of normal and injured ligaments. AJR Am J Roentgenol.

[12] Flores DV, Mejía Gómez C, Fernández Hernando M, Davis MA, Pathria MN (2019). Adult acquired flatfoot deformity: anatomy, biomechanics, staging, and imaging findings. Radiographics.

[13] Niki H, Aoki H, Inokuchi S (2005). Development and reliability of a standard rating system for outcome measurement of foot and ankle disorders I: development of standard rating system. J Orthop Sci.

[14] Niki H, Aoki H, Inokuchi S (2005). Development and reliability of a standard rating system for outcome measurement of foot and ankle disorders II: interclinician and intraclinician reliability and validity of the newly established standard rating scales and Japanese Orthopaedic Association rating scale. J Orthop Sci.

[15] Halasi T, Kynsburg A, Tállay A, Berkes I (2004). Development of a new activity score for the evaluation of ankle instability. Am J Sports Med.

[16] van Dijk CN, Reilingh ML, Zengerink M, van Bergen CJ (2010). Osteochondral defects in the ankle: why painful?. Knee Surg Sports Traumatol Arthrosc.

[17] Lassus J, Salo J, Jiranek WA (1998). Macrophage activation results in bone resorption. Clin Orthop Relat Res.

[18] Gottschalk O, Baumbach SF, Altenberger S (2021). Influence of the medial malleolus osteotomy on the clinical outcome of M-BMS + I/III collagen scaffold in medial talar osteochondral lesion (German Cartilage Register/Knorpelregister DGOU). Cartilage.

[19] Seow D, Shimozono Y, Gianakos AL, Chiarello E, Mercer N, Hurley ET, Kennedy JG (2021). Autologous osteochondral transplantation for osteochondral lesions of the talus: high rate of return to play in the athletic population. Knee Surg Sports Traumatol Arthrosc.

[20] Hurley ET, Shimozono Y, McGoldrick NP, Myerson CL, Yasui Y, Kennedy JG (2019). High reported rate of return to play following bone marrow stimulation for osteochondral lesions of the talus. Knee Surg Sports Traumatol Arthrosc.

[21] Steman JA, Dahmen J, Lambers KT, Kerkhoffs GM (2019). Return to sports after surgical treatment of osteochondral defects of the talus: a systematic review of 2347 cases. Orthop J Sports Med.

[22] van Eekeren IC, van Bergen CJ, Sierevelt IN, Reilingh ML, van Dijk CN (2016). Return to sports after arthroscopic debridement and bone marrow stimulation of osteochondral talar defects: a 5- to 24-year follow-up study. Knee Surg Sports Traumatol Arthrosc.

[23] Deland JT (2008). Adult-acquired flatfoot deformity. J Am Acad Orthop Surg.

[24] Ling SK, Lui TH (2017). posterior tibial tendon dysfunction: an overview. Open Orthop J.

[25] Bresnahan PJ, Juanto MA (2020). Pediatric flatfeet-a disease entity that demands greater attention and treatment. Front Pediatr.

[26] Tome J, Nawoczenski DA, Flemister A, Houck J (2006). Comparison of foot kinematics between subjects with posterior tibialis tendon dysfunction and healthy controls. J Orthop Sports Phys Ther.

[27] Hintermann B, Sommer C, Nigg BM (1995). Influence of ligament transection on tibial and calcaneal rotation with loading and dorsi-plantarflexion. Foot Ankle Int.

[28] Campbell KJ, Michalski MP, Wilson KJ, Goldsmith MT, Wijdicks CA, LaPrade RF, Clanton TO (2014). The ligament anatomy of the deltoid complex of the ankle: a qualitative and quantitative anatomical study. J Bone Joint Surg Am.

[29] van Eekeren IC, Reilingh ML, van Dijk CN (2012). Rehabilitation and return-to-sports activity after debridement and bone marrow stimulation of osteochondral talar defects. Sports Med.

[30] Rehman M, Duarte Silva F, Chhabra A (2024). Diagnostic efficacy of posterior tibialis tendon dysfunction: a systematic review of literature. Eur Radiol.

